# Loss and Grief Among Bereaved Family Members During COVID-19 in Brazil: A Grounded Theory Analysis

**DOI:** 10.3390/bs15060829

**Published:** 2025-06-17

**Authors:** Paola Kallyanna Guarneri Carvalho de Lima, Carlos Laranjeira, Lígia Carreira, Vanessa Denardi Antoniassi Baldissera, Viviani Camboin Meireles, Wanessa Cristina Baccon, Lashayane Eohanne Dias, Amira Mohammed Ali, Fernanda Fontes Mello, Maria Fernanda do Prado Tostes, Maria Aparecida Salci

**Affiliations:** 1 Department of Postgraduate Nursing, State University of Maringá, Avenida Colombo, 5790-Campus Universitário, Maringá 87020-900, Brazil; paolakgcl@gmail.com (P.K.G.C.d.L.); ligiacarreira.uem@gmail.com (L.C.); vanessadenardi@hotmail.com (V.D.A.B.); vivianicamboinmeireles@hotmail.com (V.C.M.); las_hayane@hotmail.com (L.E.D.); fernanda.fontesmello@gmail.com (F.F.M.); mfpprado@gmail.com (M.F.d.P.T.); masalci@uem.br (M.A.S.); 2 School of Health Sciences, Polytechnic of Leiria, Campus 2, Morro do Lena, Alto do Vieiro, Apartado 4137, 2411-901 Leiria, Portugal; 3 Centre for Innovative Care and Health Technology (ciTechCare), Polytechnic University of Leiria, Campus 5, Rua das Olhalvas, 2414-016 Leiria, Portugal; 4 Comprehensive Health Research Centre (CHRC), University of Évora, 7000-801 Évora, Portugal; 5 Departamento de Enfermagem, Unicesumar, Av. Guedner, 1610-Jardim Aclimação, Maringá 87050-900, Brazil; wanessabaccon@hotmail.com; 6 Department of Psychiatric Nursing and Mental Health, Faculty of Nursing, Alexandria University, Smouha, Alexandria 21527, Egypt; amira.mohali@alexu.edu.eg

**Keywords:** COVID-19, grounded theory, bereavement, loss, grief, family, Brazil

## Abstract

The COVID-19 pandemic has resulted in countless losses around the world, profoundly affecting the lives of many people, especially those who faced the death of family members, bringing several negative repercussions to these families and constraining the experience of grief. This study aimed to understand the experience of loss and grief among bereaved individuals who lost family members during the COVID-19 pandemic. This qualitative study was guided by Charmaz’s constructivist grounded theory as a methodological framework. The study adhered to the Criteria for REporting Qualitative research (COREQ) checklist. Data collection took place between May and November 2023 through telephone interviews that were audio-recorded and later transcribed in full. The purposive sample consisted of 21 bereaved family members who had lost their loved ones during the COVID-19 pandemic. Participants were mainly female (n = 16) with a mean age of 55.5 (SD = 16.2). The loss of their family members occurred 12 to 24 months before data collection. The following central phenomenon was identified through the analytical process: “Family experience of loss and grief: between the unspoken goodbye and post-loss adjustment”. This was anchored in the following three categories: (1) Anguish and fear of the unknown; (2) Death by COVID-19—communication of death and lack of goodbyes; and (3) (Re)construction of meaning—support networks and the grieving process. Our findings recommend that policymakers allocate additional resources to grief support services to better prepare for future pandemic events. Furthermore, it is necessary to invest in the implementation of relevant training programs for healthcare professionals, with a family centered approach.

## 1. Introduction

The COVID-19 pandemic has resulted in a global health crisis and significant loss of life, presenting unparalleled challenges to individuals, particularly in their management of loss and grief (Stroebe & Schut, 2021). During this pandemic, over 6.8 million individuals succumbed to COVID-19 globally, with an even greater number perishing from other associated causes during the same timeframe (Johns Hopkins University, 2023; Taylor, 2022). The health hazards and corresponding safety measures implemented by governments to minimize the spread of COVID-19 significantly impacted the ability of grieving families to bid farewell to their loved ones and process their loss (Stroebe & Schut, 2021).

Grief is the multifaceted human response to the loss of a loved one and the subsequent processing of that loss (Schoo et al., 2025). This represents a significant psychological transformation, involving adaptive processes and potential reconfigurations following the dissolution of an emotional attachment. Grief induces internal perceptions of security risks and requires substantial readjustments in one’s assumed reality. In this sense, a socio-ecological perspective assumes that individuals are connected to—and embedded within—a network of interpersonal, institutional, and community relationships (Neimeyer et al., 2014; Barros-Lane et al., 2024). Considering all the layers of grief, Neimeyer (2019) created a model that emphasizes meaning making in the bereavement process. He proposes that a component of the grieving experience involves deliberately creating a new narrative that incorporates the loss into one’s life story. This approach acknowledges that a component of grieving involves reconciling the past with the present and future, thereby encouraging individuals to actively confront their grief and begin to transmute their pain into wisdom and personal growth (Neimeyer et al., 2014; Neimeyer, 2019).

The COVID-19 pandemic profoundly affected the lives of many people, especially those who faced the death of family members, bringing a series of negative repercussions to these families, and making it difficult to experience and re-signify grief (Lazarova et al., 2023; Sachs et al., 2022). This changed how they perceived, experienced, and prepared for the process of death and dying (Shahini et al., 2022; Sola et al., 2023). Although the grieving process is considered a normal and necessary condition for processing loss, there are numerous specificities when facing the loss of a family member due to COVID-19 that hinder this process, including visitation restrictions; a lack of information about the health status of the hospitalized family member; the absence of funeral rituals and appropriate farewells (Asgari et al., 2022; Cost et al., 2021; Laranjeira et al., 2022a; Neimeyer & Lee, 2022; Shahini et al., 2022); and other personal and social variables, such as family support, the type of bond with the lost family member, and the emotional suffering resulting from prolonged confinement (Bosi & Alves, 2023; Sirrine et al., 2023).

Many of those who lost loved ones experienced an especially difficult grieving process, known as pandemic grief, characterized by symptoms such as death wishes, identity confusion, apathy, difficulty remembering, and lack of meaning (Sola et al., 2023; Tao et al., 2022; Walsh, 2020). Evidence indicates that difficulty or lack of participation in the farewells of loved ones increased the risk of feelings of guilt, as well as feelings of abandonment due to the perception of little social support, worsening the feeling of isolation and loneliness (Bosi & Alves, 2023; Schmidt et al., 2020; Singer et al., 2020).

Grief is part of a myriad of experiences. Therefore, evaluating the aspects of this process specifically related to the COVID-19 pandemic has become essential (Canuto et al., 2023; Dantas et al., 2020). Studies have shown that family members of people with COVID-19 experienced distress due to not knowing how the disease would progress or how they would be impacted by changes in hospital policies and facilities (Barbosa et al., 2022; Barreto et al., 2023). Furthermore, after the death of a family member, grieving individuals had to deal with remote funerals and burials, without the presence of family members or the possibility of interaction and support during the ritual (Neimeyer et al., 2006; Wallace et al., 2020).

Recent research has confirmed that grief following COVID-19 is more intense than with deaths from other causes (Eisma et al., 2021; Oluyase et al., 2021). Alarmingly, approximately two-thirds of COVID-19 bereaved individuals reported significant impairment in their ability to perform social, occupational, and family roles (Lucena et al., 2024; Osofsky et al., 2020; Sola et al., 2023). The pandemic caused psychological distress, uncertainty about the future, disruption of social support networks, economic impacts, concern for personal safety, and constant fear of illness and death among family and friends. At the organizational level, COVID-19 pandemic overburdened health systems, highlighting their structural weaknesses and lack of preparation to deal with a public health crisis of such magnitude (Araújo & Júnior, 2023; Filip et al., 2022; Gadagnoto et al., 2022). Thus, one of the major challenges that health systems faced was dealing with the overload of mental health care for those who faced the death of loved ones during the COVID-19 pandemic (Clemente-Suárez et al., 2021; Ornell et al., 2020).

A significant portion of the literature exploring grief during the COVID-19 pandemic consists of correlational, survey-based studies that analyze specific aspects of the grief experience, such as risk factors, grief reactions, and grief outcomes (Breen et al., 2022; Selman et al., 2022). This research has offered valuable insights into certain aspects of loss and grief during the COVID-19 pandemic. However, there has been limited investigation of the broad experience of bereavement in this unusual environment. Bereavement endures well after death; therefore, its study remains pertinent and essential, particularly within the Brazilian context, where qualitative studies are limited. Moreover, Brazil encountered distinct obstacles and complications compared to other regions globally, marked by elevated death rates, misinformation, the disintegration of the healthcare system, economic turmoil, and social divisions, which influenced the grief experience (Sola et al., 2024). Given the context of the research, the following question should be addressed: How did people who lost their loved ones during the COVID-19 pandemic experience loss and the bereavement process? Although COVID-19 is no longer classified as a pandemic, it has had a significant impact over a prolonged time (Sarker et al., 2023), namely for bereaved family members. In this sense, this study aimed to gain an in-depth understanding of families’ experiences of loss and grief during and after the death of their loved ones during the COVID-19 pandemic in Brazil. Furthermore, this study will provide valuable information for the development of public policies in specialized support for those bereaved by COVID-19. One can therefore understand the bereavement process, (re)organize practices and, consequently, improve the adjustment process to loss and anticipate the risk of prolonged grief.

## 2. Materials and Methods

### 2.1. Study Design

A qualitative study based on Charmaz’s constructivist grounded theory (CGT) (Charmaz, 2017) was performed. This prospective approach seeks to understand the experiences and interactions of people or groups in a given social context and obtain theoretical knowledge about psychosocial phenomena (Girardon-Perlini et al., 2020; Nascimento, 2023). Qualitative research allows bereaved persons to articulate their emotions and communicate their reflections on the significant alterations stemming from hospitalization, death, and the funeral process following the loss of their loved ones, as well as its repercussions. This study was conducted and reported following the COREQ checklist (Buus & Perron, 2020).

### 2.2. Context and Participants

The research was conducted in the southern Brazilian state of Paraná. Although this region has greater hospital resources than other areas of the country, its COVID-19 mortality rates exceeded those of locations with less adequate medical facilities (Gadagnoto et al., 2022), warranting the recruitment of individuals from this region who had lost family members to COVID-19.

The inclusion criteria were bereaved adults who lost family members to COVID-19 in the state of Paraná between November 2021 and June 2022, along with the ability to understand and communicate in Portuguese. Individuals unreachable by telephone were excluded after five unsuccessful contact attempts. Participants were recruited regardless of gender, race, or occupation, to obtain maximum sampling variation.

The number of participants was not predetermined; instead, it was the result of a deliberate and theoretical sampling process. The first twelve participants were selected according to a purposive sampling technique, and a preliminary analysis of their experiences allowed us to identify key categories or concepts (Noble & Mitchell, 2016; Patton, 2002). We started by using telephone records of individuals who developed acute COVID-19 and who had significant losses due to COVID-19. Potential participants were retrieved from the “Notifica COVID Paraná” and “Influenza Epidemiological Surveillance Information System (SIVEP-Gripe)” databases (both are information systems that monitor SARS cases, whose notification is mandatory). Theoretical sampling was subsequently employed to finalize data collection; analysis occurred concurrently to inform the selection of pertinent data and hence influence the development of the emerging theory (Charmaz, 2014). Finally, upon achieving theoretical saturation, sampling was terminated, as no additional insights were derived from the data (Charmaz, 2014). According to CGT, achieving data saturation is independent of sample size, as Charmaz (2014) articulated that saturation pertains to categories. Our final sample comprised 21 bereaved family members.

### 2.3. Data Collection

Data were gathered over six months, from June to November 2023. Eligible participants were contacted via telephone, during which the study’s objectives and the significance of their involvement were explained. Upon acceptance, the Informed Consent Form was dispatched through the participant’s preferred method (either WhatsApp or email), and the interview was arranged for a date and time specified by the participant. Data collection was carried out by a cisgender nurse with seven years of clinical experience during her PhD in Nursing. To minimize social desirability and response biases, a respectful and non-confrontational approach was adopted during the interviews. Techniques such as open-ended questions, paraphrasing, reformulation, synthesis and periods of silence were used, encouraging participants to express their feelings. Furthermore, we sought to build adequate rapport through active listening, demonstrating empathy and establishing a relationship grounded in trust.

The interviews lasted between 30 and 50 min (average of 40 min). They were audio-recorded and subsequently transcribed in full. Interviews were conducted based on a semi-structured script, developed to fulfil the study objectives and supported by the available literature (Mason et al., 2020; Sola et al., 2023; Tao et al., 2022). The open-ended questions addressed “the history of the relationship with the deceased relative”, “the experience of loss”, “the evolution and elaboration of the grieving process”, and the “socio-emotional support received (friends, family and health services), after the loss”. The script was validated by three researchers with experience in the subject, with slight adjustments to the wording of the questions. There were no repetitions or follow-up interviews.

### 2.4. Data Analysis

Data collection and analysis were conducted concurrently, employing a constant comparative method during this process (Bingham, 2023; Dennis et al., 2022). This enabled the researchers to consistently concentrate on advancing and refining notions derived from the data (Pauli et al., 2022). Initially, the data were coded inductively, line by line. This was followed by targeted coding to elucidate connections among categories. The final phase of coding was theoretical integration, which evolved from the initiation of the analytical process until all categories were saturated, substantiating the phenomenon. The categories were structured around a basic notion known as the core category. This explanatory model arose from the researcher’s capacity to integrate theoretical sensitivity with the inductive–deductive process to formulate the theory elucidating the phenomenon under investigation (Fife & Gossner, 2024; Padgett, 2017; Santos et al., 2018). Data were administered, saved, and analyzed utilizing MaxQDA^®^ 24 software (Rädiker & Kuckartz, 2019). Sample quotations were organized according to the sequence of interviews (e.g., P1…, P21) and age.

### 2.5. Rigor and Reflexivity

Charmaz offered the following four criteria to ensure the correctness and validity of research: credibility, originality, resonance, and usefulness (Charmaz & Thornberg, 2020). The interviews were meticulously transcribed, and field notes were consistently compared and verified to ensure the study’s authenticity. Credibility was established using the following three methods: member checking, prolonged involvement, and peer debriefing (Charmaz, 2014; Nowell et al., 2017). Member checking is a procedure wherein participants meticulously assess and authenticate the precision of the findings, increasing the degree of confirmability (Charmaz, 2014; Dado et al., 2023). Prolonged involvement allows researchers to discern recurring patterns and themes that may not be evident during brief interactions. Peer debriefing involves obtaining feedback from colleagues or experts to validate interpretations and reduce researcher bias (Charmaz, 2014; Dado et al., 2023). The originality of the findings was confirmed through an extensive reflection process involving memo writing and literature evaluation, alongside diagramming approaches executed by the project team (Charmaz, 2014, 2017; Nowell et al., 2017). The discussion echoes the need to contextualize the findings within prior research and to evaluate applicability to analogous individuals and circumstances. Given that this research was grounded in the constructivist paradigm, the researcher’s reflexivity significantly influenced the data discovery process. Uniqueness and significance of the findings were achieved through interviews (conducted until data saturation) and the application of the constant comparative approach to validate developing information. The continuous comparison of the coded notions produced the primary category, discerned through an iterative procedure. The MaxQDA^®^ software facilitates data organization and generates a comprehensive roadmap for a qualified auditor to assess the entire process (Rädiker & Kuckartz, 2019). Analysis was carried out by the primary author, who regularly engaged in discussions with the co-authors utilizing memos and diagrams (utility criteria). Participant quotations were translated into English for publication purposes, and the authors bear responsibility for them, as the original language was Portuguese. All team members have prior experience in qualitative research and are aligned with the constructivist epistemological framework.

### 2.6. Ethics

The study protocol was approved by the State University of Maringá—UEM Research Ethics Committee (Opinion No. 4214589). Written informed consent (including permission to audio-record the responses) was given for each interview. Participants were informed that they could choose to withdraw from the study at any time. They were not compensated for their participation. Strategies were implemented to address participant distress by offering immediate assistance from the interviewer and directing participants to professional bereavement support services.

## 3. Results

### 3.1. Participants Background

The sample included 21 people, aged between 27 and 84 years (55.5 ± 16.2). Participants were predominantly female (n = 16; 76.2%) and white (n = 15; 71.4%). Regarding education, the majority of participants had less than eight years of study (n = 12; 57.1%). All participants lost one or more family members due to COVID-19, with nine reporting the loss of a parent, six reporting the loss of a spouse, six reporting the loss of an uncle and/or cousin, three reporting the loss of a sibling, and one participant reporting the loss of a grandmother. The loss of family members occurred 12 to 24 months before the interview. Table 1 presents a summary description of the characteristics of the study participants.

### 3.2. Interview Findings

**Figure 1:**
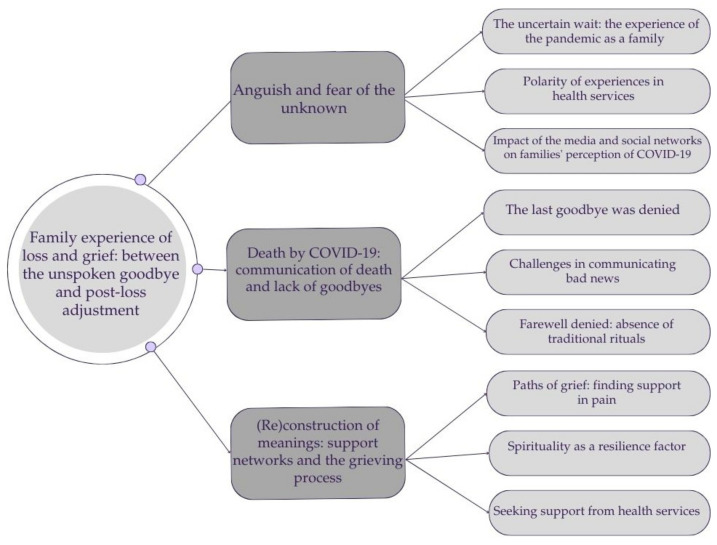
Categories and subcategories of central phenomenon: “Family experience of loss and grief: between the unspoken goodbye and post-loss adjustment”.

Through the analytical process, the following central phenomenon was identified: “Family experience of loss and grief: between the unspoken goodbye and post-loss adjustment”. This was anchored in three categories and nine subcategories that support the phenomenon and help us to understand the findings (Figure 1). The uncertainty associated with the COVID-19 trajectory, the hospitalization process of the sick family member, and the redefinition after loss reflect the complexity of the process of loss and mourning experienced by bereaved family members.

The three identified categories were as follows: (1) Anguish and fear of the unknown; (2) Death by COVID-19: communication of death and lack of goodbyes; and (3) (Re)construction of meanings: support networks and the grieving process.

#### 3.2.1. Anguish and Fear of the Unknown

This category refers to COVID-19 as an infectious disease that has caused numerous deaths and hospitalizations and, consequently, generated negative emotions that are difficult to manage. At the same time, it integrates the heterogeneity of experiences in service provision, as well as the impact of social networks on the intensification of family fear and anguish.

The first subcategory—the uncertain wait: the experience of the pandemic as a family—portrays the sudden disruption experienced in the family context, which determined the reconfiguration of the family’s status quo. Participants highlighted the need to seek new paths from an individual but also collective perspective, having to adapt to new routines and the measures imposed by social isolation.

*[…] For me, COVID-19 had a huge impact. I quit my job, gave up my apartment, a radical change […] all because of COVID-19. I stopped working and stayed at my father’s house taking care of him because he had become infected. I had very difficult days because there was no way for him to receive the medical care he needed*.

(P9, 54 years old)

*[…] He was isolated in a room, in a bathroom, and I stayed in the other rooms of the house with our dogs. I wore gloves, a mask, alcohol gel, separated his cutlery from mine, and when I took them out I had to wash them separately. He also took great care to ensure that I didn’t touch him, so as not to get infected*.

(P18, 46 years old)

*[…] We were scared, yes. When the infection appeared, we started monitoring my father and uncle’s saturation, and the values were always low. We realized that keeping them at home was no longer possible. It was a difficult decision, because the hospitals were full*.

(P20, 28 years old)

The COVID-19 experience affected the family nucleus for all participants. Not only did they have to reinvent family dynamics, adapting to a “new normal,” they also had to deal with the guilt of being vehicles of infection for their deceased loved ones.

*[…] My wife, 36 years together, and she passed away from COVID-19. In fact, I caught COVID and I was the one who passed it on to her*.

(P2, 59 years old)

*[…] COVID was very hard on my family. My mother has COPD and was the first to become infected. My father, because he was worried about her, took care of her. However, as he had heart problems and diabetes, he ended up catching the infection and ended up dying. Sometimes I think that I should have been more careful and not let him take care of my mother*.

(P20, 28 years old)

In most cases, the unpredictability of the course of the disease did not allow for any prior planning, particularly regarding the sick family member’s preferences and wishes.

*[…] It was very quick, he felt ill and went to look for a basic health unit. Meanwhile, he left by ambulance for the hospital […] it all happened very quickly. It was not possible to prepare anything*.

(F21, 43 years old)

Only P5 reported having the opportunity to validate with the sick family member their wishes regarding the end of life: *[…] I stayed talking, me and him, until three in the morning. And he said everything whether I knew he was an organ donor, if I knew he wanted to be buried here and wanted to be cremated, so, he said everything, three days later he passed away* (P5, 79 years old).

The second subcategory—polarity of experiences in health services—reflects the ambivalence in the care received during the pandemic, highlighting the quality of care. Some participants reported positive experiences in which healthcare professionals played a vital role in helping them get through one of the most difficult times in their lives—dealing with the severity of their family members’ COVID-19 illness.

*[…] The people who helped us best were the nurses and physiotherapists, these people provided good care, were well prepared. Days after my mother passed away, I went to the hospital and took a basket to the staff as a sign of gratitude. They were doing their best there, without a doubt*.

(P16, 36 years old)

*[…] She [sister] was very well looked after, because every day they came to see her, everyone helped, someone always gave support and cheered for her*.

(P12, 41 years old)

*[…] Everyone blames doctors, not me, I praise them. And I think people have to stop with this ignorance, they did everything they could to save people, including risking their own lives*.

(F18, 46 years old)

In contrast, some participants highlighted that care in hospitals was problematic due to the lack of organization and the precariousness of health services. The shortage of vaccines, the lack of available beds, and the extreme overload of professionals and hospital units were factors that generated fear, anguish, and intense suffering for the family members involved.

*[…] They were the most difficult and darkest days of my life, because we even fought for a private ICU. If that were the case, I would sell what I had to save his [husband’s] life, but there was no vacancy. I know what I’m going to say is strong, but they had to choose who would live and who would die*.

(P18, 46 years old)

*[…] At the time, there was no ICU available, he [brother] stayed in the hospital and got better. However, the disease ended up affecting the lungs, kidneys and other organs. If he had the care he needed, perhaps he wouldn’t have died*.

(P14, 55 years old)

*[…] They set up makeshift ICUs. There was always someone with the curtain closed, someone who had passed away and was waiting for their family, I ended up witnessing all that suffering of the families*.

(P21, 43 years old)

The third subcategory—impact of the media and social networks on families’ perception of COVID-19—portrays the strong impact generated by the news and the fear it caused among participants. Political denialism regarding the severity of the pandemic’s effects also generated distrust and dismay.

*[…] She [wife] stayed at home, well we both had COVID-19, but she was much worse than me, she had trouble breathing and didn’t feel well. And then the news on TV drove us crazy… there was a lot of misleading news, I remember seeing a news story that recommended taking ivermectin. I almost ruined my health by having to take this medicine. Wow, it was horrible, right? But the worst, the worst was the media*.

(P2, 59 years old)

*[…] It was very difficult. On television, the president joked that he was not a gravedigger, he imitated COVID-19 patients, that monster. There is no greater pain in the world than losing someone unjustly. What happened in Brazil and in the world, but in Brazil specifically, is collective murder*.

(P18, 46 years old)

Although misinformation was generated during the pandemic, the media did positively influence people’s perception of their health, particularly regarding the importance of social isolation and the proper use of protective equipment and hygiene care. In addition, it reported news about hospitalizations and deaths, which, for some people, raised greater awareness about the severity of the virus and the need to protect their families.

*Although the news was not the best, television warned people to be cautious. We saw the news about the mortality associated with COVID-19 and we ended up not leaving the house to protect ourselves*.

(P18, 46 years old)

*I redoubled my care with the use of a mask and hygiene to protect those I loved*.

(P13, 32 years old)

#### 3.2.2. Death by COVID-19: Communication of Death and Lack of Goodbyes

This category reveals the burden of the absence of farewells, both the final farewell with the family member and the traditional rituals after death. COVID-19 prevented grieving family members from processing their losses, creating traumatic experiences. The pandemic context generated intense pain and uncertainty in the family, amplifying the feeling of unreality (especially due to the lack of farewell) and making it difficult to come to terms with the loss of a loved one.

The first subcategory—the last goodbye was denied—highlights findings that demonstrate the challenges when experiencing the loved one’s hospitalization, marked by restrictions on visits, difficulties in receiving information, and by the desire to provide a final goodbye. The denied goodbye began with the safety measures taken as soon as the sick family member was hospitalized and prevented from maintaining physical contact with the outside world: a separation that became definitive. The experience of being denied a final goodbye can have emotional, psychological and social impacts.

*[…] They only let me and my brother go there to visit, just once. She [mother] was unconscious and intubated. For us it was a farewell, because the following morning she passed away. My other relatives didn’t have the opportunity to say goodbye*.

(P16, 36 years old)

*[…] When she [sister] went there, it was the worst thing, because we didn’t see her anymore and we couldn’t even talk on the phone. That was the worst part, if I had known that those were her last days, I would have left her at home to die with her family*.

(P12, 41 years old)

*[…] We went to the ICU and only saw her through the glass [mother]. We couldn’t talk to her, and there we said a prayer, we said goodbye to her, because we knew she was really going. And that night she died*.

(P17, 59 years old)

Faced with the impossibility of accompanying their family members to the hospital, having regular visits, or being present in their final moments, some family members reported the close care from professionals, allowing them to establish contact with their loved ones through the use of mobile devices.

*[…] It was very important because I recorded audios, they took his [husband’s] hand, passed on the audios of our godchildren, who are like children to us, and he got very emotional*.

(P18, 46 years old)

*[…] At first, my mother used WhatsApp to send us photos, with the mask on, but when it was time to remove the oxygen, I was very short of breath. Then, when it got worse, the nurses made the call, because she was already intubated*.

(P16, 36 years old)

The second subcategory—challenges in communicating bad news—portrays the lack of preparation for the death of family members, whether due to lack of information, the sometimes depersonalized way information was transmitted over the telephone, or because it was not addressed to the designated family member. The impossibility of being present during the last moments and the long-distance communication intensified feelings of shock and helplessness, leaving emotional scars that influenced the grieving process.

*[…] They called me the next morning when my brother and I went to visit, they just said that my mother had passed away. To this day I still don’t believe it, because during the visit no one warned us that the situation was very serious and that there was a risk of death. We were not prepared for what happened*.

(F16, 36 years old)

*[…] The professional called my mother at one twenty in the morning and said that my brother had just died. Luckily, she wasn’t alone. But receiving news like that, without preparation, brought her to her knees. I had asked the nurse on duty to call me*.

(F14, 55 years old)

*[…] She [sister] was there (in the hospital) and we were here isolated. When this bad news came, I knew she had been hospitalized, but when she got worse, I didn’t know, because for me she was fine and suddenly she wasn’t fine anymore. My God, I miss her so much, I miss her so much. I still feel her with me…*.

(F1, 50 years old)

The third subcategory—farewell denied: absence of traditional rituals—describes how difficult it was for grieving family members not to be by their dying loved ones’ side, to be unable to perform farewell rituals, and to lack significant others’ support due to the imposed restrictions. These difficulties contributed negatively to the quality of the death experience and the grieving process.

*[…] There were no goodbyes, we couldn’t say goodbye, there was nothing… It was just a black bag. At that time, no one buried anyone, we couldn’t hold wakes, we couldn’t do anything. To this day I blame myself for this*.

(P1, 50 years old)

*[…] The coffin was sealed, we didn’t see him, we don’t know if it was him [husband]. I sent clothes, but they said they didn’t need clothes and threw them away, that time was the worst… How could we say goodbye if we didn’t see him. I still think it might not have been him…*

(P3, 78 years old)

*[…] On the day of the funeral, my mother and I were in the car. I had symptoms, and my mother had COVID. It was very difficult, and it left us with scars. My father simply left home on Sunday, and my mother says that it feels like he left, that he abandoned us. A horrible feeling, because you are with the person here, the person leaves and never comes back*.

(P9, 55 years old)

*[…] When my mother passed away, they all came with her in the funeral car. They had barely lowered the window when they said, “You are Mrs. Catarina’s relatives.” We said, “Yes,” and they said, “Then get in.” When we arrived at the grave, there was a tape, like when someone is murdered, they put tape around it, so people wouldn’t get too close. We were so focused on that, that they then put her inside, covered it, and left. When I looked around, there was no one else there. I said, we forgot to say a farewell prayer, it was very shocking*.

(P17, 59 years old)

Although the final farewell was absent for most interviewed families, there were cases of family members who managed to carry out alternative rituals, such as a burial transmitted by video call, or even holding a funeral procession as a form of memorialization. Death rituals help to contextualize the loss in time and space, facilitating the reorganization of roles and the continuity of life. In addition, they offer cultural and emotional support to the family when facing the numbness and confusion caused by the loss.

*[…] The most that could be done was a car parade in a funeral procession, which brought together some family members, each in their own car*.

(F13, 32 years old)

*[…] Since his family couldn’t come, we did a video call broadcast on Instagram so the family could watch. My oldest son didn’t want to go, so he stayed at home with my mother. She said that he attended the funeral and kept pressing the little heart, the white heart on the live stream kept rising*.

(F21, 43 years old)

#### 3.2.3. (Re)Construction of Meanings: Support Networks and the Grieving Process

This category reveals the post-loss mourning trajectory, during which bereaved family members seek to make sense of the new reality in the absence of their loved one. This process is experienced in a unique and individual way, involving a renegotiation of one’s own identity, as well as new forms of engagement with the outside world.

The first subcategory—paths of grief: finding support in pain—describes how the support of friends and family was essential in this process. Through the narratives, we realize that the support of friends and family played a crucial role in helping the bereaved during this period of suffering. The solidarity shown by those closest to us strengthens emotional bonds and helps to mitigate the pain of loss.

*[…] If I didn’t have my friends, if I didn’t, I would really want to throw myself in front of a train. I spent about six months where nothing mattered to me, nothing. […] My friends gave me a lot of support. Wow, I have a friend here who would travel to come see me, to see how I was doing, he would call me every day. If it weren’t for my friends, I don’t know where I would be, what would become of me now*.

(P2, 59 years old)

*[…] Grief is eternal, we just get used to the pain and that’s why support from others is important. There were a lot of people who were left alone, that’s much worse*

(P4, 55 years old)

Good memories of those who have passed away are recognized as a way to aid the grieving process, giving loved ones the strength to move forward. They help preserve the presence of those who have passed away. Photographs, videos, and other visual mementos serve as emotional anchors, allowing individuals to revisit happy moments and feel connected to the people they have loved.

*[…] I would call my mother to tell her about everything good that happened in my life. Even today, whenever something happens, I say, I have to call my mother, then I remember that she’s not here anymore, but then I look at the photographs and it helps me*.

(P17, 59 years old)

*[…] We talked, we laughed, we worked, she helped me here at home. It was little, but she helped me, I miss her. I remember with joy when we would have coffee together, drink chimarrão [tea] and I feel that she is close to me*.

(P10, 68 years old)

The second subcategory—spirituality as a resilience factor—portrays the impact of participants’ connection with the divine on overcoming adversity and their ability to find meaning in life. Spirituality provides people with a sense of purpose and connection to something greater, offering meaning to the pain of loss. In addition, spirituality fosters hope and helps create community support networks, which emotionally strengthen those facing the challenge of grief.

*[…] Despite all the horror, all the difficulties, God helps me*.

(P21, 43 years old)

*[…] We do what God asks of us, we all have a purpose. God takes care of me and this helps me find the strength to take care of others*.

(P19, 68 years old)

*[…] I think this way, that God knows what he does, it was his time. COVID-19 was just the circumstance. So, we have to think this way, otherwise we suffer too much*.

(P9, 50 years old)

The third subcategory—seeking support from health services—describes the difficulties experienced by bereaved family members in accessing specialized grief support. These difficulties also included the lack of post-mortem follow-up by services, effectively “forgetting” those who lost loved ones to COVID-19.

*[…] I went to the health center and asked the doctor if there was a psychiatrist or psychologist who could help me. She said she there was none, and didn’t know if there would be*.

(P20, 28 years old)

*[…] They didn’t offer support, nothing, everyone is left in the dark*.

(P17, 59 years old)

Two participants added that participating in this study gave them the opportunity to speak openly about their concerns for the first time, and that these moments were therapeutic in themselves.

*[…] No support, no one in the hospital, no one, nothing. You were the only one to call me. We had nothing, not a word, nothing*.

(P10, 68 years old)

*[…] I think there was a lack of support from the government and there was a lot of fuss. Anyway, it was missing. I think the Unified Health Service [SUS] is great, but there was a lack of good service, but we understand that there was a pandemic. In fact, there was only you, only you who came to talk to me about COVID-19, that’s why I made a point of talking to you, what you’re doing is very important*.

(P2, 59 years old)

Nevertheless, some participants reported positive experiences with support provided by health professionals and services.

*[…] I had support from Unimed (Health Plan), social assistance, psychologist, everything. Unimed is very well supported. The psychologist, when he passed away, came to see me, she prepared me a lot, it was very important to have talked to her*.

(P7, 65 years old)

*[…] When my mother passed away, the hospital had a psychologist, and I was asked if I, my brother or my father needed any support and that ended up happening*.

(P16, 36 years old)

## 4. Discussion

This qualitative study investigated the experiences of bereaved family members during and following the loss of loved ones due to COVID-19. The findings reveal difficult experiences faced by bereaved family members during the pandemic. The isolation measures imposed affected family members’ ability to cope with loss and grief due to the lack of physical contact with loved ones before or at the time of death. Family members suffered not only from the difficulties of dealing with the loss of a loved one during isolation but also from the loneliness experienced caused by the inability to receive support from health professionals (Gesi et al., 2020; Vedder et al., 2022). Similarly, evidence suggests that people were deprived of the opportunity to pay tribute to their loved ones, leading to concerns that they would pass on alone, without receiving the warmth of the people they love (Kokou-Kpolou et al., 2020). Almost all types of social interaction were prohibited, such as funerals and burials. In addition, the participants felt hopelessness and uncertainty about the progression of the pandemic. The lack of preparation for death gave rise to a shared sense of lack of purpose and uncertainty, as well as insufficient time for the grieving person to process the loss (Cost et al., 2021; Olufadewa et al., 2020; Smid, 2020).

Due to the various losses suffered throughout the COVID-19 pandemic, the grieving process has become a critical aspect to consider, given its impact on the psychological health of those in mourning (Chen, 2020; Diolaiuti et al., 2021). While social distancing reduced the positive effects of social support, spirituality assumed a facilitating role in the grieving process by allowing the attribution of meanings (van Schaik et al., 2022). Several studies have recognized the emotional and psychological impact of coping with grief during a pandemic, as well as with the difficulties in accessing support, which intensified the negative emotions associated with grief and loss (Harrop et al., 2022, 2023). Death due to COVID-19 contributed to the experience of loss as a traumatic event that, combined with a lack of previous experience in coping with grief, amplified the risk of prolonged grief (Blackburn et al., 2023; Eisma & Tamminga, 2022; Liang et al., 2022; Simbi et al., 2020). Also, the lack of support—due to the social isolation measures imposed during the pandemic—prevented the farewell practices and rituals normally associated with loss and mourning, intensifying negative emotions and impeding the ability to cope with the situation (Firouzkouhi et al., 2023; Fuller et al., 2021). In this regard, using concept analysis, Costeira et al. (2024) introduced the concept of death unpreparedness, which generated negative consequences for grieving family members during the pandemic. The core attributes of the concept encompass uncertainty and unpredictability due to the virus’s erratic activity, the swift deterioration causing separation anxiety, and the inability to satisfy desires and end-of-life preferences, resulting in alterations to final dying rituals. This transition typically manifests in the following two contrasting manners: (1) post-traumatic growth and acceptance of death, or (2) a maladaptive grieving process with a significant risk of prolonged grief (Costeira et al., 2024).

In the current study, it was observed that the family members were rarely allowed to say goodbye to their loved ones, and they also reported dissatisfaction with the circumstances of the death. The findings further show that some family members were dissatisfied and did not accept the death of their loved ones, even though they were informed about the condition of the sick family member by telephone or video (Kumar, 2021; Wakam et al., 2020). Furthermore, funeral rituals—which usually bring relief after death—were performed without the presence of relatives, friends, and family, or were conducted virtually, negatively impacting the bereaved (Cunha et al., 2023; Dantas et al., 2020; Pimenta & Capelas, 2020).

The pandemic introduced unforeseen and unparalleled obstacles in society, with healthcare professionals encountering moral and ethical dilemmas during this arduous period (Iserson, 2020). Menon and Padhy (2020) reported that physicians faced circumstances requiring them to select patients for ventilator allocation. They emphasized that such decisions should be predicated on the likelihood of survival rather than the need for resources, which may be accompanied by a diminished chance of survival (Menon & Padhy, 2020). Relatives frequently learned about these healthcare issues via the media, instead of obtaining the necessary updates regarding their family member’s status or condition at the time of death (Ummel et al., 2022). As one of the countries most affected by the pandemic, Brazil also faced challenges related to political denialism, which encouraged suspicious or indifferent behaviors toward the social value of science (Guimarães & Moreira, 2022). The findings highlight the then-president Bolsonaro’s denialism, as he encouraged non-compliance with protective measures and vaccination. This resulted in Brazil having one of the highest COVID-19 fatality rates and, consequently, the highest numbers of bereaved families.

The findings underscore the significance of public programs that encompass holistic care, ranging from socio-community initiatives to specialized support for grieving individuals. Comprehending the nuances of the grieving experience during the COVID-19 pandemic is crucial for formulating and implementing effective treatment strategies (Lana et al., 2020). The relevance of this issue led the World Health Organization to consider Prolonged Grief Disorder in the International Classification of Diseases. It is therefore necessary to optimize the capacity to intervene in situations of complex and persistent grief, helping to ensure that all citizens in need have access to specialized care in the prevention and treatment of prolonged grief. The early identification of physical and psychological suffering is important, as it can protect against the development of chronic conditions in those affected (Laranjeira & Querido, 2021; Mortazavi et al., 2023).

### 4.1. Study Limitations

This study employed qualitative interviews, yielding a more profound understanding and closer examination of the participants’ actual experiences. However, this study has significant limitations, including the use of a convenience sample of bereaved family members recruited solely via telephone. Individuals lacking access to this technology could have addressed additional themes that represent other real experiences of these communities. Moreover, telephone interviews present a potential bias due to the absence of nonverbal communication cues. Nonetheless, we underscore that its utilization facilitated access to a cohort of participants from various regions in the southern part of the country. Data transferability to other Brazilian states is restricted, as the effects of COVID-19 have varied significantly. The impact of the COVID-19 pandemic on ritual performance differed across different regions and countries. In certain contexts, bereaved families were permitted to conduct smaller in-person rituals, yet in other instances, social rituals following death were significantly restricted or entirely forbidden (van Schaik et al., 2022). Bereaved relatives who engaged in ritual acts subsequently acknowledged that the memorial provided solace by facilitating post-traumatic growth following a loss (Carson et al., 2023; Hernández-Fernández & Meneses-Falcón, 2021; van Schaik et al., 2022). Moreover, the sample population predominantly consisted of females who self-identified as white, yet experiences may vary for individuals from other cultural groups with distinct gender distributions. In this vein, it is crucial to acknowledge how racism affects access to grieving support, promoting the importance of ethno-racial specific community circles of care (Reece, 2020). Furthermore, the participants’ memories of previous experiences may be influenced by recall bias. Lastly, data analysis neglected variations depending on the time of death, such as the length of grief or the existence of various restrictions.

### 4.2. Implications for Practice

In the post-pandemic world, it is essential for health and social care institutions, along with their professionals, to derive insights from the experiences of individuals who suffered bereavement during the pandemic, in order to reconstruct integrated and compassionate services, while also contemplating preparedness for future analogous occurrences. Intervening in bereavement due to COVID-19 presupposes a reorganization of practices and, concomitantly, improvements in the care model and the organization of health services to meet this health demand. Thus, this study could contribute to the qualification of health services and promote improvements in care for all—based on scientific evidence that can support decision-making in health policies in the short, medium, and long term (Hinkson et al., 2024; Morais et al., 2024). Additional investment is needed to increase the availability of personalized grief support services to effectively address the needs of those who have experienced loss and to mitigate the phenomena of prolonged grief (Giamattey et al., 2021). This includes providing culturally sensitive and competent crisis care, as well as implementing group-based support programs that cater to individuals with common experiences and characteristics. To proactively engage communities and raise awareness of available support options, it is recommended that information on available bereavement support services be provided (Vitorino et al., 2024). A set of strategies are proposed, including immediate application or long-term support. Immediate interventions could be applied in future pandemic scenarios and could include telephone and video calls, audio recordings, written messages, and the use of personal objects in hospital beds, such as photos and music (Estrela et al., 2021). These strategies could help compensate for the restriction of visits and promote benefits, such as reducing the feeling of isolation among patients and facilitating communication between staff, patients, and family members, contributing to a more humanized interaction in hospital environments (Feder et al., 2021; Mateus et al., 2024).

Long-term strategies include individual or group psychological care, remote farewell techniques, the expansion of the psychosocial care network to monitor people with signs of depression or prolonged grief disorder (Estrela et al., 2021; Laranjeira et al., 2022b), and the strengthening of religious and/or spiritual networks (Biancalani et al., 2022; Laranjeira & Querido, 2023). These strategies have the potential to help family members say goodbye to those who have passed away and support each other—even if virtually in some cases—leading to significant impacts on the social dimension of death and dying (Cherblanc et al., 2025; Fernández & González-González, 2022). Social media has also influenced public displays of grief. The integration of digital media into daily life has led to social networking sites being regarded as suitable venues for expressing grief and commemorating the deceased. Under certain circumstances, specialized social media platforms can be established to commemorate the deceased, allowing users to exchange experiences and fostering a sense of co-presence with other individuals and groups in mourning (Usher & Jackson, 2023). In parallel, policymakers should allocate additional resources to grief support services to better prepare for future pandemic events. Furthermore, it is necessary to invest in implementing training programs for healthcare professionals to improve the quality of community-based bereavement support.

## 5. Conclusions

The study’s findings underscore the effects of the COVID-19 pandemic on family members who have experienced the loss of a loved one. Significant loss and enduring grief were reported by bereaved family members whose last contacts were limited by pandemic constraints. Notwithstanding the considerable sacrifices endured, families conveyed compliance for the sake of public health and solidarity with the medical personnel attending to their relatives. The COVID-19 pandemic has affected families’ grieving experiences with adverse effects on their functioning and psychological well-being. This justifies the need to promote cultural and organizational efforts to raise awareness among the population and create psychological intervention protocols aimed at those bereaved by COVID-19. This study also emphasizes the integration of bereavement care into future pandemic planning to reduce unmet needs, while also advocating for the enhancement of professional skills and the expansion of evidence-based bereavement services (i.e., for psychotherapies addressing processes that are directly pertinent to Prolonged Grief Disorder) without pathologizing normative grief. Furthermore, healthcare professionals and public health authorities should use these family-informed viewpoints when developing crisis protocols and future administrative regulations to enhance end-of-life care for hospitalized patients.

## Figures and Tables

**Table 1:** Description of sample of family members bereaved by COVID-19.

Participants	Age	Sex	Race	Education (Years)	Number of Losses to COVID-19	Type of Bond with Deceased
P1	50	Male	White	<8	One	Mother
P2	59	Male	White	≥8	One	Spouse
P3	78	Female	White	<8	One	Spouse
P4	55	Female	White	<8	Two	Mother/Sister
P5	79	Female	White	<8	One	Son
P6	30	Female	White	≥8	One	Grandmother
P7	65	Female	White	<8	One	Spouse
P8	84	Female	White	<8	One	Spouse
P9	54	Female	White	≥8	One	Father
P10	68	Female	Non-white	<8	One	Mother
P11	56	Male	Non-white	<8	One	Mother
P12	41	Female	Non-white	≥8	Two	Sister/Cousin
P13	32	Female	White	≥8	Three	Uncle/Aunt/Cousin
P14	55	Male	White	<8	One	Brother
P15	40	Female	Non-white	≥8	One	Mother
P16	36	Male	White	≥8	One	Mother
P17	59	Female	White	<8	One	Mother
P18	46	Female	White	≥8	One	Spouse
P19	68	Female	Non-white	<8	One	Cousin
P20	28	Female	Non-white	≥8	Two	Father/Uncle
P21	43	Female	White	≥8	One	Spouse

## Data Availability

This paper is a part of the doctoral thesis of the first author, and all data generated or analyzed during this study are included in this article.

## References

[B1-behavsci-15-00829] Araújo J. I., Júnior G. A. (2023). Bereaved families: The process of elaborating grief as a result of death from COVID-19. Scientia Generalis.

[B2-behavsci-15-00829] Asgari Z., Naghavi A., Abedi M. R. (2022). Beyond a traumatic loss: The experiences of mourning alone after parental death during COVID-19 pandemic. Death Studies.

[B3-behavsci-15-00829] Barbosa T. D., Melo M. S. S., Menezes D. A. (2022). Analysis of family grief in the context of COVID-19: An integrative review. Research, Society and Development.

[B4-behavsci-15-00829] Barreto M. D. S., Marques F. R. D. M., Gallo A. M., Garcia-Vivar C., Carreira L., Salci M. A. (2023). Striking a new balance: A qualitative study of how family life has been affected by COVID-19. Revista Latino-Americana de Enfermagem.

[B5-behavsci-15-00829] Barros-Lane L., Germany A., Smith P., Stovall T. (2024). A socioecological examination of the challenges associated with young widowhood: A systematic review. Omega.

[B6-behavsci-15-00829] Biancalani G., Azzola C., Sassu R., Marogna C., Testoni I. (2022). Spirituality for coping with the trauma of a loved one’s death during the COVID-19 pandemic: An Italian qualitative study. Pastoral Psychology.

[B7-behavsci-15-00829] Bingham A. J. (2023). From data management to actionable findings: A five-phase process of qualitative data analysis. International Journal of Qualitative Methods.

[B8-behavsci-15-00829] Blackburn J., Waring G., Turner M., Currell K., Caress A. L. (2023). Exploring the impact of bereavement during the COVID-19 pandemic on children and young people: A scoping review. Comprehensive Child and Adolescent Nursing.

[B9-behavsci-15-00829] Bosi M. L. M., Alves E. D. (2023). Social distancing in urban contexts in the COVID-19 pandemic: Challenges for the field of mental health. Physis: Revista de Saúde Coletiva.

[B10-behavsci-15-00829] Breen L. J., Mancini V. O., Lee S. A., Pappalardo E. A., Neimeyer R. A. (2022). Risk factors for dysfunctional grief and functional impairment for all causes of death during the COVID-19 pandemic: The mediating role of meaning. Death Studies.

[B11-behavsci-15-00829] Buus N., Perron A. (2020). The quality of quality criteria: Replicating the development of the Consolidated Criteria for Reporting Qualitative Research (COREQ). International Journal of Nursing Studies.

[B12-behavsci-15-00829] Canuto R. M. S., Ferreira A. C. L., Novaes L. F., Salles R. J. (2023). The grieving process in relatives of COVID-19 victims. Studies and Research in Psychology.

[B13-behavsci-15-00829] Carson J., Gunda A., Qasim K., Allen R., Bradley M., Prescott J. (2023). Losing a loved one during the COVID-19 pandemic: An on-line survey looking at the effects on traumatic stress, coping and post-traumatic growth. Omega.

[B14-behavsci-15-00829] Charmaz K. (2014). Constructing grounded theory: A practical guide through qualitative analysis.

[B15-behavsci-15-00829] Charmaz K. (2017). Constructivist grounded theory. The Journal of Positive Psychology.

[B16-behavsci-15-00829] Charmaz K., Thornberg R. (2020). The pursuit of quality in grounded theory. Qualitative Research in Psychology.

[B17-behavsci-15-00829] Chen R. (2020). Social support as a protective factor against the effect of grief reactions on depression for bereaved single older adults. Death Studies.

[B18-behavsci-15-00829] Cherblanc J., Côté I., Mercure C., Boever C., Zech E. (2025). Rituals in grief: Why meaning matters more than numbers. Journal of Loss and Trauma.

[B19-behavsci-15-00829] Clemente-Suárez V. J., Martínez-González M. B., Benitez-Agudelo J. C., Navarro-Jiménez E., Beltran-Velasco A. I., Ruisoto P., Diaz Arroyo E., Laborde-Cárdenas C. C., Tornero-Aguilera J. F. (2021). The impact of the COVID-19 pandemic on mental disorders. A critical review. International Journal of Environmental Research and Public Health.

[B20-behavsci-15-00829] Cost K. T., Crosbie J., Anagnostou E., Birken C. S., Charach A., Monga S., Kelley E., Nicolson R., Maguire J. L., Burton C. L., Schachar R. J., Arnold P. D., Korczak D. J. (2021). Mostly worse, occasionally better: Impact of COVID-19 pandemic on the mental health of Canadian children and adolescents. European Child & Adolescent Psychiatry.

[B21-behavsci-15-00829] Costeira C., Dixe M. A., Querido A., Rocha A., Vitorino J., Santos C., Laranjeira C. (2024). Death unpreparedness due to the COVID-19 pandemic: A concept analysis. Healthcare.

[B22-behavsci-15-00829] Cunha M., Simões G., Soares J., Santos E. (2023). Grief and coping in family members and significant others of people who died from COVID-19. Journal of Nursing Referência.

[B23-behavsci-15-00829] Dado M., Spence J. R., Elliot J. (2023). The case of contradictions: How prolonged engagement, reflexive journaling, and observations can contradict qualitative methods. International Journal of Qualitative Methods.

[B24-behavsci-15-00829] Dantas C. d. R., Azevedo R. C. S. d., Vieira L. C., Côrtes M. T. F., Federmann A. L. P., Cucco L. d. M., Rodrigues L. R., Domingues J. F. R., Dantas J. E., Portella I. P., Cassorla R. M. S. (2020). Grief in times of COVID-19: Challenges of care during the pandemic. Latin American Journal of Fundamental Psychopathology.

[B25-behavsci-15-00829] Dennis B., Vanstone M., Swinton M., Brandt Vegas D., Dionne J. C., Cheung A., Clarke F. J., Hoad N., Boyle A., Huynh J., Toledo F., Soth M., Neville T. H., Fiest K., Cook D. J. (2022). Sacrifice and solidarity: A qualitative study of family experiences of death and grief in intensive care settings during the pandemic. BMJ Open.

[B26-behavsci-15-00829] Diolaiuti F., Marazziti D., Beatino M. F., Mucci F., Pozza A. (2021). Impact and consequences of COVID-19 pandemic on complicated grief and persistent complex bereavement disorder. Psychiatry Research.

[B27-behavsci-15-00829] Eisma M. C., Tamminga A. (2022). COVID-19, natural, and unnatural bereavement: Comprehensive comparisons of loss circumstances and grief severity. European Journal of Psychotraumatology.

[B28-behavsci-15-00829] Eisma M. C., Tamminga A., Smid G. E., Boelen P. A. (2021). Acute grief after deaths due to COVID-19, natural causes and unnatural causes: An empirical comparison. Journal of Affective Disorders.

[B29-behavsci-15-00829] Estrela F. M., Silva A. F., Oliveira A. C. B., Magalhães J. R. F., Soares C. F. S., Peixoto T. M., Oliveira M. A. S. (2021). Coping with mourning for family loss due to COVID-19: Short and long-term strategies. Persona y Bioética.

[B30-behavsci-15-00829] Feder S., Smith D., Griffin H., Shreve S. T., Kinder D., Kutney-Lee A., Ersek M. (2021). “Why couldn’t I go in to see him?” Bereaved families’ perceptions of end-of-life communication during COVID-19. Journal of the American Geriatrics Society.

[B31-behavsci-15-00829] Fernández Ó., González-González M. (2022). The dead with no wake, grieving with no closure: Illness and death in the days of coronavirus in Spain. Journal of Religion and Health.

[B32-behavsci-15-00829] Fife S. T., Gossner J. D. (2024). Deductive qualitative analysis: Evaluating, expanding, and refining theory. International Journal of Qualitative Methods.

[B33-behavsci-15-00829] Filip R., Gheorghita Puscaselu R., Anchidin-Norocel L., Dimian M., Savage W. K. (2022). Global challenges to public health Care systems during the COVID-19 pandemic: A review of pandemic measures and problems. Journal of personalized medicine.

[B34-behavsci-15-00829] Firouzkouhi M., Alimohammadi N., Abdollahimohammad A., Bagheri G., Farzi J. (2023). Bereaved families views on the death of loved ones due to COVID 19: An integrative review. Omega.

[B35-behavsci-15-00829] Fuller J. A., Hakim A., Victory K. R., Date K., Lynch M., Dahl B., Henao O., CDC COVID-19 Response Team (2021). Mitigation policies and COVID-19-associated mortality—37 European countries, January 23–June 30, 2020. MMWR. Morbidity and Mortality Weekly Report.

[B36-behavsci-15-00829] Gadagnoto T. C., Mendes L. M. C., Monteiro J. C. d. S., Gomes-Sponholz F. A., Barbosa N. G. (2022). Emotional consequences of the COVID-19 pandemic in adolescents: Challenges to public health. Revista da Escola de Enfermagem da USP.

[B37-behavsci-15-00829] Gesi C., Carmassi C., Cerveri G., Carpita B., Cremone I. M., Dell’Osso L. (2020). Complicated grief: What to expect after the coronavirus pandemic. Frontiers in Psychiatry.

[B38-behavsci-15-00829] Giamattey M. E. P., Frutuoso J. T., Bellaguarda M. L. d. R., Luna I. J. (2021). Funeral rituals in the COVID-19 pandemic and mourning: Possible reverberations. Anna Nery School.

[B39-behavsci-15-00829] Girardon-Perlini N. M. O., Simon B. S., Lacerda M. R. (2020). Grounded theory methodological aspects in Brazilian nursing thesis. Revista Brasileira de Enfermagem.

[B40-behavsci-15-00829] Guimarães R. M., Moreira M. R. (2022). How does the context effect of denialism reinforce the oppression of the vulnerable people and negatively determine health? Lancet Regional Health. Americas.

[B41-behavsci-15-00829] Harrop E., Goss S., Longo M., Seddon K., Torrens-Burton A., Sutton E., Farnell D. J., Penny A., Nelson A., Byrne A., Selman L. E. (2022). Parental perspectives on the grief and support needs of children and young people bereaved during the COVID-19 pandemic: Qualitative findings from a national survey. BMC Palliative Care.

[B42-behavsci-15-00829] Harrop E., Medeiros Mirra R., Goss S., Longo M., Byrne A., Farnell D. J. J., Seddon K., Penny A., Machin L., Sivell S., Selman L. E. (2023). Prolonged grief during and beyond the pandemic: Factors associated with levels of grief in a four time-point longitudinal survey of people bereaved in the first year of the COVID-19 pandemic. Frontiers in Public Health.

[B43-behavsci-15-00829] Hernández-Fernández C., Meneses-Falcón C. (2021). I can’t believe they are dead. Death and mourning in the absence of goodbyes during the COVID-19 pandemic. Health & Social Care in the Community.

[B44-behavsci-15-00829] Hinkson G. M., Huggins C. L., Doyle M. (2024). Transnational caregiving and grief: An autobiographical case study of loss and love during the COVID-19 pandemic. Omega.

[B45-behavsci-15-00829] Iserson K. V. (2020). Healthcare ethics during a pandemic. The Western Journal of Emergency Medicine.

[B46-behavsci-15-00829] Johns Hopkins University (2023). COVID-19 dashboard.

[B47-behavsci-15-00829] Kokou-Kpolou C. K., Fernández-Alcántara M., Cénat J. M. (2020). Prolonged grief related to COVID-19 deaths: Do we have to fear a steep rise in traumatic and disenfranchised griefs?. Psychological Trauma: Theory, Research, Practice and Policy.

[B48-behavsci-15-00829] Kumar R. M. (2021). The many faces of grief: A systematic literature review of grief during the COVID-19 pandemic. Illness, Crisis & Loss.

[B49-behavsci-15-00829] Lana R. M., Coelho F. C., Gomes M. F. d. C., Cruz O. G., Bastos L. S., Villela D. A. M., Codeço C. T. (2020). Emergence of the new coronavirus (SARS-CoV-2) and the role of timely and effective national health surveillance. Cadernos de Saúde Pública.

[B50-behavsci-15-00829] Laranjeira C., Moura D., Marcon S., Jaques A., Salci M. A., Carreira L., Cuman R., Querido A. (2022a). Family bereavement care interventions during the COVID-19 pandemic: A scoping review protocol. BMJ Open.

[B51-behavsci-15-00829] Laranjeira C., Moura D., Salci M. A., Carreira L., Covre E., Jaques A., Cuman R. N., Marcon S., Querido A. (2022b). A scoping review of interventions for family bereavement care during the COVID-19 pandemic. Behavioral Sciences.

[B52-behavsci-15-00829] Laranjeira C., Querido A. (2021). Changing rituals and practices surrounding COVID-19 related deaths: Implications for mental health nursing. British Journal of Mental Health Nursing.

[B53-behavsci-15-00829] Laranjeira C., Querido A. (2023). An in-depth introduction to arts-based spiritual healthcare: Creatively seeking and expressing purpose and meaning. Frontiers in Psychology.

[B54-behavsci-15-00829] Lazarova M., Caligiuri P., Collings D. G., De Cieri H. (2023). Global work in a rapidly changing world: Implications for MNEs and individuals. Journal of World Business.

[B55-behavsci-15-00829] Liang N., Becker T. D., Rice T. (2022). Preparing for the COVID-19 paediatric mental health crisis: A focus on youth reactions to caretaker death. Clinical Child Psychology and Psychiatry.

[B56-behavsci-15-00829] Lucena P. L. C., Alves A. M. P. d. M., Batista P. S. d. S., Agra G., Lordão A. V., Costa S. F. G. d. (2024). End-of-life care and grief: A study with family members of COVID-19 victims. Ciência & Saúde Coletiva.

[B57-behavsci-15-00829] Mason T. M., Tofthagen C. S., Buck H. G. (2020). Complicated grief: Risk factors, protective factors, and interventions. Journal of Social Work in End-of-Life & Palliative Care.

[B58-behavsci-15-00829] Mateus M. J., Simões L., Ali A. M., Laranjeira C. (2024). Family experiences of loss and bereavement in palliative care units during the COVID-19 pandemic: An interpretative phenomenological study. Healthcare.

[B59-behavsci-15-00829] Menon V., Padhy S. K. (2020). Ethical dilemmas faced by health care workers during COVID-19 pandemic: Issues, implications and suggestions. Asian Journal of Psychiatry.

[B60-behavsci-15-00829] Morais J., Arruda G., Melo C. d. F., Martins C. (2024). Real and symbolic grief experiences during COVID-19 pandemic: An integrative literature review: A. Portuguese Journal of Behavioral and Social Research.

[B61-behavsci-15-00829] Mortazavi S. S., Shahbazi N., Taban M., Alimohammadi A., Shati M. (2023). Mourning during corona: A phenomenological study of grief experience among close relatives during COVID-19 pandemics. Omega.

[B62-behavsci-15-00829] Nascimento R. (2023). Meaning versus sense: A proposal for a conceptual distinction to studies on grief. Studies and Research in Psychology.

[B63-behavsci-15-00829] Neimeyer R. A. (2019). Meaning reconstruction in bereavement: Development of a research program. Death Studies.

[B64-behavsci-15-00829] Neimeyer R. A., Baldwin S. A., Gillies J. (2006). Continuing bonds and reconstructing meaning: Mitigating complications in bereavement. Death Studies.

[B65-behavsci-15-00829] Neimeyer R. A., Klass D., Dennis M. R. (2014). A social constructionist account of grief: Loss and the narration of meaning. Death Studies.

[B66-behavsci-15-00829] Neimeyer R. A., Lee S. A. (2022). Circumstances of the death and associated risk factors for severity and impairment of COVID-19 grief. Death Studies.

[B67-behavsci-15-00829] Noble H., Mitchell G. (2016). What is grounded theory?. Evidence-Based Nursing.

[B68-behavsci-15-00829] Nowell L. S., Norris J. M., White D. E., Moules N. J. (2017). Thematic analysis: Striving to meet the trustworthiness criteria. International Journal of Qualitative Methods.

[B69-behavsci-15-00829] Olufadewa I. I., Adesina M. A., Oladokun B., Baru A., Oladele R. I., Iyanda T. O., Abudu F. (2020). “I was scared i might die alone”: A qualitative study on the physiological and psychological experience of COVID-19 survivors and the quality of care received at health facilities. International Journal of Travel Medicine and Global Health.

[B70-behavsci-15-00829] Oluyase A. O., Hocaoglu M., Cripps R. L., Maddocks M., Walshe C., Fraser L. K., Preston N., Dunleavy L., Bradshaw A., Murtagh F. E. M., Bajwah S., Sleeman K. E., Higginson I. J., CovPall study team (2021). The challenges of caring for people dying from COVID-19: A multinational, observational study of palliative and hospice services (CovPall). Journal of Pain and Symptom Management.

[B71-behavsci-15-00829] Ornell F., Schuch J. B., Sordi A. O., Kessler F. H. P. (2020). “Pandemic fear” and COVID-19: Mental health burden and strategies. Brazilian Journal of Psychiatry.

[B72-behavsci-15-00829] Osofsky J. D., Osofsky H. J., Mamon L. Y. (2020). Psychological and social impact of COVID-19. Psychological Trauma: Theory, Research, Practice and Policy.

[B73-behavsci-15-00829] Padgett D. K. (2017). Qualitative methods in social work research.

[B74-behavsci-15-00829] Patton M. Q. (2002). Two decades of developments in qualitative inquiry: A personal, experiential perspective. Qualitative Social Work.

[B75-behavsci-15-00829] Pauli B., Strupp J., Schloesser K., Voltz R., Jung N., Leisse C., Bausewein C., Pralong A., Simon S. T. (2022). It’s like standing in front of a prison fence—Dying during the SARS-CoV2 pandemic: A qualitative study of bereaved relatives’ experiences. Palliative Medicine.

[B76-behavsci-15-00829] Pimenta S., Capelas M. (2020). Intervention in the grief process in Portugal by palliative care teams. Cadernos De Saúde.

[B77-behavsci-15-00829] Rädiker S., Kuckartz U. (2019). Analyse qualitativer daten mit MAXQDA.

[B78-behavsci-15-00829] Reece R. (2020). A reflection on racial injustice and (black) anticipatory grief compounded by COVID-19. Journal of Concurrent Disorders.

[B79-behavsci-15-00829] Sachs J. D., Karim S. S. A., Aknin L., Allen J., Brosbøl K., Colombo F., Barron G. C., Espinosa M. F., Gaspar V., Gaviria A., Haines A., Hotez P. J., Koundouri P., Bascuñán F. L., Lee J. K., Pate M. A., Ramos G., Reddy K. S., Serageldin I., Michie S. (2022). The lancet commission on lessons for the future from the COVID-19 pandemic. Lancet.

[B80-behavsci-15-00829] Santos J. L. G. D., Cunha K. S., Adamy E. K., Backes M. T. S., Leite J. L., Sousa F. G. M. (2018). Data analysis: Comparison between the different methodological perspectives of the Grounded Theory. Revista da Escola de Enfermagem da USP.

[B81-behavsci-15-00829] Sarker R., Roknuzzaman A. S. M., Hossain M. J., Bhuiyan M. A., Islam M. R. (2023). The WHO declares COVID-19 is no longer a public health emergency of international concern: Benefits, challenges, and necessary precautions to come back to normal life. International Journal of Surgery.

[B82-behavsci-15-00829] Schmidt B., Crepaldi M. A., Bolze S. D. A., Neiva-Silva L., Demenech L. M. (2020). Mental health and psychological interventions during the new coronavirus pandemic (COVID-19). Estudos de Psicologia (Campinas).

[B83-behavsci-15-00829] Schoo C., Azhar Y., Mughal S., Rout P. (2025). Grief and prolonged grief disorder. StatPearls [Internet].

[B84-behavsci-15-00829] Selman L. E., Farnell D., Longo M., Goss S., Seddon K., Torrens-Burton A., Mayland C. R., Wakefield D., Johnston B., Byrnem A., Harrop E. (2022). Risk factors associated with poorer experiences of end-of-life care and challenges in early bereavement: Results of a national online survey of people bereaved during the COVID-19 pandemic. Palliative Medicine.

[B85-behavsci-15-00829] Shahini N., Abbassani S., Ghasemzadeh M., Nikfar E., Heydari-Yazdi A. S., Charkazi A., Derakhshanpour F. (2022). Grief experience after deaths: Comparison of COVID-19 and non-COVID-19 causes. Journal of Patient Experience.

[B86-behavsci-15-00829] Simbi C. M. C., Zhang Y., Wang Z. (2020). Early parental loss in childhood and depression in adults: A systematic review and meta-analysis of case-controlled studies. Journal of Affective Disorders.

[B87-behavsci-15-00829] Singer J., Spiegel J. A., Papa A. (2020). Pre-loss grief in family members of COVID-19 patients: Recommendations for clinicians and researchers. Psychological Trauma: Theory, Research, Practice, & Policy.

[B88-behavsci-15-00829] Sirrine E. H., Kliner O., Gollery T. J. (2023). College student experiences of grief and loss amid the COVID-19 global pandemic. Journal of Death and Dying.

[B89-behavsci-15-00829] Smid G. E. (2020). A framework of meaning attribution following loss. European Journal of Psychotraumatology.

[B90-behavsci-15-00829] Sola P. P. B., Santos M. A., Oliveira-Cardoso É. A. (2024). Emotional suffering after the COVID-19 pandemic: Grieving the loss of family members in Brazil. International Journal of Environmental Research and Public Health.

[B91-behavsci-15-00829] Sola P. P. B., Souza C., Rodrigues E. C. G., Santos M. A. D., Oliveira-Cardoso É. A. (2023). Family grief during the COVID-19 pandemic: A meta-synthesis of qualitative studies. Cadernos de Saude Publica.

[B92-behavsci-15-00829] Stroebe M., Schut H. (2021). Bereavement in times of COVID-19: A review and theoretical framework. Omega.

[B93-behavsci-15-00829] Tao X., Yu C. C., Low J. (2022). Exploring loss and grief during the COVID-19 pandemic: A scoping review of qualitative studies. Annals Singapore.

[B94-behavsci-15-00829] Taylor L. (2022). COVID-19: True global death toll from pandemic is almost 15 million, says WHO. BMJ.

[B95-behavsci-15-00829] Ummel D., Vachon M., Guité-Verret A. (2022). Acknowledging bereavement, strengthening communities: Introducing an online compassionate community initiative for the recognition of pandemic grief. American Journal of Community Psychology.

[B96-behavsci-15-00829] Usher K., Jackson D. (2023). Public expressions of grief and the role of social media in grieving and effecting change. International Journal of Mental Health Nursing.

[B97-behavsci-15-00829] van Schaik T., Brouwer M. A., Knibbe N. E., Knibbe H. J. J., Teunissen S. C. C. M. (2022). The effect of the COVID-19 pandemic on grief experiences of bereaved relatives: An overview review. Omega.

[B98-behavsci-15-00829] Vedder A., Boerner K., Stokes J. E., Schut H. A. W., Boelen P. A., Stroebe M. S. (2022). A systematic review of loneliness in bereavement: Current research and future directions. Current Opinion in Psychology.

[B99-behavsci-15-00829] Vitorino J. V., Duarte B. V., Ali A. M., Laranjeira C. (2024). Compassionate engagement of communities in support of palliative and end-of-life care: Challenges in post-pandemic era. Frontiers in Medicine.

[B100-behavsci-15-00829] Wakam G. K., Montgomery J. R., Biesterveld B. E., Brown C. S. (2020). Not dying alone—Modern compassionate care in the COVID-19 pandemic. The New England Journal of Medicine.

[B101-behavsci-15-00829] Wallace C. L., Wladkowski S. P., Gibson A., White P. (2020). Grief during the COVID-19 pandemic: Considerations for palliative care providers. Journal of Pain and Symptom Management.

[B102-behavsci-15-00829] Walsh F. (2020). Loss and resilience in the time of COVID-19: Meaning making, hope, and transcendence. Family Process.

